# Understanding of mental fatigue in elite fencing sports: perspectives from Chinese national level fencers

**DOI:** 10.3389/fpsyg.2025.1512326

**Published:** 2025-04-14

**Authors:** Chao Bian, Suzanna Russell, Kevin De Pauw, Jelle Habay, Špela Bogataj, Bart Roelands

**Affiliations:** ^1^Human Physiology and Sports Physiotherapy Research Group, Faculty of Physical Education and Physiotherapy, Vrije Universiteit Brussel, Brussels, Belgium; ^2^Sports Performance, Recovery, Injury and New Technologies Research Centre (SPRINT), Faculty of Health Sciences, Australian Catholic University, Brisbane, QLD, Australia; ^3^Performance Services, Australian Institute of Sport, Bruce, ACT, Australia; ^4^Sport Performance Innovation and Knowledge Excellence (SPIKE), Queensland Academy of Sport, Nathan, QLD, Australia; ^5^BruBotics, Vrije Universiteit Brussel, Brussels, Belgium; ^6^Vital Signs and Performance Monitoring Research Unit, LIFE Department, Royal Military Academy, Brussels, Belgium; ^7^Research Foundation Flanders (FWO), Brussels, Belgium; ^8^Department of Nephrology, University Medical Centre Ljubljana, Ljubljana, Slovenia; ^9^Faculty of Sport, University of Ljubljana, Ljubljana, Slovenia

**Keywords:** cognitive fatigue, qualitative study, combat sports, epee, foil, sabre

## Abstract

**Introduction:**

Recent studies have documented the presence, fluctuation, and persistence of mental fatigue (MF) across various elite sports. It appears that open-skilled competitive contexts tend to impose greater mental demands, leading to higher levels of MF. Fencing, as an open-skilled combat sport, requires perceptual-cognitive skills and mental resources allocation for an optimal performance. However, it is underrepresented in the MF research domain.

**Methods:**

This study employed a cross-sectional design using an online survey to capture Tier 3–5 Chinese fencers’ perceptions of MF and their general understanding of the contributors that may induce MF in fencing. Descriptive reports, thematic analysis, comparisons of retrospective MF perceptions (MVAS) and different contributors to MF inducement in training and competition were conducted.

**Results:**

The results highlight a contextual difference of MF (training vs. competition), and the contributions of timing-related decision-making moments and execution of offensive actions to the MF inducement in fencing. Furthermore, MF was perceived higher in the direct elimination stage competition than in the pool stage (MVAS: 57.6 ± 21.0 vs. 49.2 ± 21.7 AU). Individuals’ health conditions and competition-oriented elements were rated as synergistic factors of MF perception, while external commitments were rated lower.

**Discussion:**

These findings emphasize the presence of MF in fencers and encourage researchers and practitioners to assess and deliberately manage MF. Future studies should involve longitudinal, multifactor observations on diverse fencers and contexts to validate current findings, with a focus on specific in-match scenarios to enhance the representativeness and inform targeted training and management strategies for MF in fencing.

## 1 Introduction

Mental fatigue is a psychobiological state induced by prolonged cognitive activity. It is characterized by a subjective feeling of tiredness, reduced cognitive capacity, and altered brain activation (Boksem and Tops, 2008). Laboratory-based studies suggest that mental fatigue impacts subsequent physical performance, psychomotor performance including decision-making ability, reaction speed, and accuracy, as well as key aspects of technical and tactical performance (Habay et al., 2021). Possible mechanisms underlying these negative mental fatigue effects are linked to complex neural mechanisms, motivation, and resource depletion (e.g., brain phosphocreatine) (Habay et al., 2021). Over the past decade, mental fatigue has received increased significant attention, especially in the context of elite and professional sports (Roelands et al., 2021). Researchers have increasingly utilized accessible tools, such as self-reported scales and questionnaires, to monitor mental fatigue longitudinally in elite athletes. Studies have documented the presence, fluctuation, and temporal nature of mental fatigue across various sports contexts, including team sports [i.e., football (Abbott et al., 2020; Thompson et al., 2021; Díaz-García et al., 2023)], netball (Russell et al., 2022a,b), rugby (Mariano et al., 2023), beach volleyball (Da Costa et al., 2023), padel (Diaz-Garcia et al., 2021), and orienteering (Lam et al., 2023). Despite the accumulating body of research, the variations, specificity, and nature of differing applied contexts make it challenging to draw broader conclusions to generalize across sports. However, open-skilled competitive contexts, which require rapid responses to unpredictable and dynamic environments in competitions, tend to impose greater mental demands and thus result in higher levels of mental fatigue compared to some closed-skilled sports (Coyne et al., 2021).

Fencing is an open-skilled combat sport that encompasses three main disciplines, categorized by the type of sword: epee, foil, and sabre (Roi and Bianchedi, 2008). Regardless of fencing gender, the average duration of a single combat bout is 17.9 ± 3.1 s for epee, 5.8 ± 2.5 s for foil, and 1.7 ± 0.4 s for sabre, with corresponding work-to-rest ratios of 1:0.9, 1:2.6, and 1:9.2, respectively (Tarragó et al., 2023). Typically, an international match day (lasting between 9 and 11 h) for a single fencing discipline includes a preliminary round (i.e., pool stage, lasting up to 3 min or first to five touches) and direct elimination rounds (up to 9 min each or first to 15 touches), with a total effective combat time ranging from 17 to 48 min (Roi and Bianchedi, 2008). The high intermittency and specific movement patterns inherent in fencing reduce anaerobic contributions during a match (Yang et al., 2022), leading to non-maximal physiological demands (Roi and Bianchedi, 2008) and relatively minor physical fatigue, as indicated by physiological (Varesco et al., 2023) and biochemical measures (Turner et al., 2017).

Despite its moderate physical demands, fencing is a highly technical and tactical sport that imposes significant cognitive load (Varesco et al., 2023). Continuous attention and rapid information processing are essential for analyzing and selecting visual cues from the opponent during fencing combat (Varesco et al., 2023; Varesco et al., 2024). Combined with making precise decisions, fencers must execute fast reactions and superior response inhibition, requiring excellent coordination and body control to achieve both speed and accuracy in their most appropriate movements within extremely short timeframes (Roi and Bianchedi, 2008; Gutiérrez-Davila et al., 2019). Furthermore, emotional regulation is highly demanded during fencing competitions due to frequent interruptions and switching between winning and losing situations throughout the stressful bouts (Doron and Martinent, 2021). It remains unclear whether the prolonged, yet intermittent, interactions involving multiple perceptual, motor, and cognitive demands in fencing (Bagot et al., 2023) lead to mental fatigue, and how this might impact specific performance capacity. However, given findings in prior sports demonstrating impacts on performance aspects important to fencing, i.e., tactical errors, slower reaction, reduced attack accuracy (Habay et al., 2021), it is reasonable to propose a potential impact. Furthermore, evidence supporting impaired decision-making ability previously found in boxing athletes following artificially induced mental fatigue (Fortes et al., 2023), demonstrates relevance to sports of a similar structure.

Primary evidence suggests that the gradual subjective manifestation of fatigue in simulated fencing competitions is linked to a significantly increased mental demand (Varesco et al., 2023). Accordingly, fatigue in real-world fencing may also predominantly result from the mental demand component. However, this has not been established yet. Given the relatively unexplored nature of this topic, especially the scarcity of quantitative mental fatigue research in fencing sports, qualitative methods provide a valuable approach to gather initial insights and establish foundational knowledge (Harper and McCunn, 2017). These qualitative findings were selected to provide a strong base to inform and guide the relevance and ecologically validity of future studies. Therefore, the purpose of this study was to design and implement a bespoke online survey for national fencing-level practitioners, aiming to investigate fencers’ perceptions of mental fatigue and their general understanding of the factors that may induce mental fatigue in fencing.

## 2 Methods

### 2.1 Study design

This study employed a cross-sectional design using an anonymous online survey. Given the survey targeted a specific fencing community, it was initially developed by the primary researchers in English but was administered in the participants’ native language, to ensure clarity and accurate expression of intent. A 10-person expert panel assisted in piloting, and provided feedback from which the survey was refined and adjusted to ensure appropriate length, structure, and clarity of questioning, prior to the final distribution. Only participants who voluntarily agreed to participate and provided informed consent were included. The project was conducted in accordance with the principles outlined in the Declaration of Helsinki and received ethical approval from Scientific Research Ethics Committee of Shanghai University of Sport (No. 102772023RT058).

### 2.2 Participants and inclusion criteria

The study used purposeful sampling to provide opportunity to include Chinese national or international level [i.e., Tier 3–5 (McKay et al., 2021)] fencing athletes across nationwide training centers and camps. Participants consisted of active fencers, as well as retired fencers with equivalent athletic levels who concurrently held team coaching, assistant, or management roles. Referees were excluded from the analysis. Eligible participants were required to be over 18 years old and in good physical and mental health determined by the absence of current injury and medication. Participants were encouraged to respond the survey individually in a neutral emotional and cognitive state.

### 2.3 Data collection

The survey was administrated via the Chinese Sojump online platform^1^ and was accessible on participants personal electronic devices through a sharable digital link. For inclusion of response in the data set, respondents’ informed consent and completion of all survey questions were required. The conditional formatting applied in the online system prohibited next-page navigation, skipping questions, or background reading to encourage quality completion that was representative of the participants’ current perception. Completion time and platform identifier code were automatically recorded for validity check. The online link to the survey was distributed by an expert fencing panel, Chinese Fencing Association, and Fencing Academy of China.

### 2.4 Survey design, pilot, and finalization

Given the exploratory nature of the bespoke, cross-sectional survey, and the targeted high-level fencer’s distinct feelings might fluctuate over time due to the changing environment and state, only content validity was considered (Anyadike-Danes et al., 2023). The theoretical framework for defining the workload factors contributing to the mental fatigue inducement in fencing was grounded on the concepts of internal/external load and fatigue (Halson, 2014). The decomposition of crucial moments in fencing bouts, considered as potential contributors to mental fatigue from a temporal perspective, was supported by time-motion analyses in foil, sabre (Aquili et al., 2013), and epee (Yang et al., 2022). The motion schemes of fencing experts also provided important reference.

The three researchers (CB, SR, BR), with both mental fatigue and sports domain expertise, designed the general survey constructs and logic in English. A bilingual researcher (CB) then translated the survey into Chinese on the platform and assembled an expert panel (*n* = 10) for two rounds of piloting and refinement. The panel included two coaches, one administrator, and three active fencers from the national fencing academy with undergraduate or above education level, one mentor from collegiate fencing teaching group, one performance director of the national fencing team, one leading lecturer of the national Foil coaching courses, and one sports science professor. They first reviewed and modified the draft independently, checked the clarity and the intention of every item, and evaluated content including fencing specificity. The pilot feedback was compiled and incorporated into a revised version of the survey. To ensure the revisions provided the desired clarity, an online meeting was held between pilot members and the lead author to resolve differing suggestions and reach unanimous approval through a public review and feedback process. This version was then finalized and activated for distribution.

### 2.5 Survey construction and details

The survey was organized into five sections, presented across five pages: an initial page for informed consent, a page outlining the background of the mental fatigue concept in sports, and three pages containing 17 questions. The original survey has been uploaded as a Supplementary Material. The response formats included single-select, multi-select, one open-ended answer, and visual analog scale (VAS) sliding matrixes. The order of options within the multiple-select and the VAS matrix were presented in a randomized manner, as allocated by the platform’s conditional logic. The survey structure covered several key areas: Basic information (identity, fencing experience); Perceptions of mental fatigue (general attitude toward mental fatigue, retrospective self-reported perception of mental fatigue (MVAS) in different fencing contexts, descriptions of mental fatigue in fencing, and synergistic influence factors on mental fatigue); and Understanding the mental fatigue inducement process and contributors (examining the impact of five general workload factors - physical, technical, tactical, psychological, and environmental loads in the training and competition contexts; and six critical combat moments - posture preparation, step movement, timing decision-making, attack, defense, and riposte execution in fencing bouts). Participants responded to the multiple contributors via presentation of a VAS matrix, with each rated by sliding each individual VAS to indicate its relative contribution.

### 2.6 Data analysis

All original answers were exported from the platform in an Excel (Version 16.71, Microsoft, Redmond, U.S.) file. The completion duration and unique participant identifier was screened for potential duplication. All complete responses were included in the data corpus for analysis. Descriptive analyses were conducted in Excel. The mean with standard deviation (± SD) was presented for quantitative variables, while the response percentage (%) among all valid responses was reported for single- or multi-select items. The median value (i.e., 50 AU) of the digital VAS full scale range was defined as the cut-off for moderate-level fatigue perception (Nordin et al., 2016) when interpreting the retrospective MVAS results.

Further analysis was conducted in SPSS (Version 25, IBM, Chicago, U.S.). The Kolmogorov–Smirnov normality test was run to check and confirmed normal distributions for the MVAS outcomes. The MVAS were compared across contexts by paired t-test. Cohen’s d effect size was calculated in case of significant difference between two contexts, with conventional thresholds applied: 0.20 (small), 0.50 (medium), and 0.80 (large). The perceived contribution data from one VAS matrix, which included five different workload factors during training, five workload factors during competition, and six combat moments in fencing bouts, were normalized to a total of 100%. In cases where some contribution rates followed a non-normal distribution, the Wilcoxon signed-rank tests were applied to compare all contributors within the matrix and between the different contexts (i.e., training and competition). The significance level was set at *p* < 0.05 (two-tailed).

NVivo (Version 12, Lumivero, Denver, U.S.) software was used to code the original texts generated from the open-ended descriptions of mental fatigue in fencing. The software was also utilized to analyze and connect the codes to establish higher themes, define and name themes, and interpret the answers, which followed the thematic analysis guidelines (Braun and Clarke, 2006). Due to the bilingual context, the primary codes were extracted in Chinese for better understanding and more accurate translation by the lead researcher (CB). Following subsequent translation to English the summarization and construction of concepts, cross-checking of the translated codes, and allocation to themes, was undertaken by two researchers independently (CB and SR). Following this, the obtained outcomes were translated back into Chinese for a final inspection of alignment with the original responses in the participants’ native language. The back-and-forth translations process followed Weiler et al. (2024) in the bilingual environment.

## 3 Results

The online link to the survey remained open for a total of eight weeks, across two periods; mid-August to mid-September 2023, and the October 2023. The data collection period deliberately avoided the potential interference of the major international fencing event, the Asian Games. The survey received a total of 102 responses, of which 92 valid responses were included in the final analysis. Seven practitioners who only identified as fencing referees or staff without indicating systematic fencing training experience were excluded on the basis of lack of fencing-specific expertise. Another three respondents who chose “not at all” in the ability to differentiate mental fatigue from physical fatigue were excluded to minimize data that was not representative of perceptions and opinions of those who indicated to hold insight into the concept of mental fatigue. The average time to complete the survey was 7.3 ± 4.8 min per valid respondent.

### 3.1 Participant demographics

Responders were female (37.0%) and male (63.0%) adult fencing athletes or team practitioners with the Tier 3–5 profiles across all three disciplines. As shown in the Table 1, the majority were 18–25-year-old (91.3%), Tier 3 (60.9%) active fencers (76.1%). Thirty-four had Tier 4 profiles, and two belonged to Tier 5.

**Table 1:** Demographic information of age, gender, fencing identity, fencing discipline, athlete tier, and years of experience in athletes.

Age/years		Gender		Identity		Discipline		Athletic level		Experience/years	
18–25	91.3%	Female	37.0%	Active	76.1%	Epee	33.7%	Tier 3	60.9%	Career	10.0 ± 4.6
26–30	3.3%	Male	63.0%	Retired	23.9%	Foil	34.8%	Tier 4	36.9%	Provincial	6.8 ± 5.4
31–40	3.3%	Others	0.0%	Coach	12.0%	Sabre	28.2%	Tier 5	2.2%	National	2.2 ± 2.8
41–50	2.2%			Others	4.3%	Undefined	3.3%			International	1.2 ± 2.2

Presents as percentage of total participants and fencing experience presents in mean ± standard deviation.

### 3.2 Perception of mental fatigue in fencing

Regarding the attitudes toward the mental fatigue, more than half (57.6%) of fencers indicated difficulty in consistently distinguishing between mental and physical fatigue after fencing practice. In addition to the provided mental fatigue definition, developed from perceptions across other sports, 62 respondents contributed additional phrases to describe mental fatigue in fencing. Twenty-three (37.1%) respondents directly stated experience of the above-normal fatigue. They highlighted that mental fatigue happened in the match-related contexts. The mental fatigue in fencing was generally associated with negative emotional, psychomotor, and bodily responses (see Table 2). It was also perceived to cause subsequent sleep disturbance.

**Table 2:** Thematic analysis presenting participants’ perceptions of feelings associated with mental fatigue in fencing.

Theme	Main code	Frequency (*n* = 62)	Percentage
Emphasis the term	Very tired	23	37.1%
Match-related context	During match	19	30.6%
	Unsatisfied results	12	19.4%
	After match	7	11.3%
Emotional response	Disgusted	16	25.8%
	Annoyed	5	8.1%
	Stressful	5	8.1%
	Anxious	4	6.5%
Psychomotor response	Disengaging thought	13	21.0%
	Blank mind	9	14.5%
	Recalling	6	9.7%
	Decreased attention	4	6.5%
	Slow reaction	3	4.8%
Somatic response	Headache	16	25.8%
	Powerless body	6	9.7%
Sleep disturbance	Drowsiness	6	9.7%
	Insomnia	3	4.8%

Based on previous fencing experience, participants recalled and reported the MVAS with 47.6 ± 18.7 AU in a typical comprehensive training session, with 49.2 ± 21.7 AU in the pool stage on an official match day with equivalent opponents, while the perception rated 57.6 ± 21.0 AU in the direct elimination stage was significantly higher than in the pool stage (*t* = 4.14, Cohen’s d = 0.39, *p* < 0.001). All fencers acknowledged the negative effects of mental fatigue on performance and health. They identified sleep quality (78.3%), match environment (50.0%), preparation duration (43.5%), diet (41.3%), and emotion (38.0%) might increase mental fatigue alongside fencing practice, followed by other factors such as interpersonal relationships (31.5%), non-fencing work (29.4%), academic requirements (23.9%), family ties (21.7%), transportation (12.0%), and media (6.5%). A high number of participants (84.8%) indicated that the integration of mental fatigue targeted training could also induce positive effects on fencing performance.

### 3.3 Contributors to mental fatigue inducement in fencing

**Figure 1:**
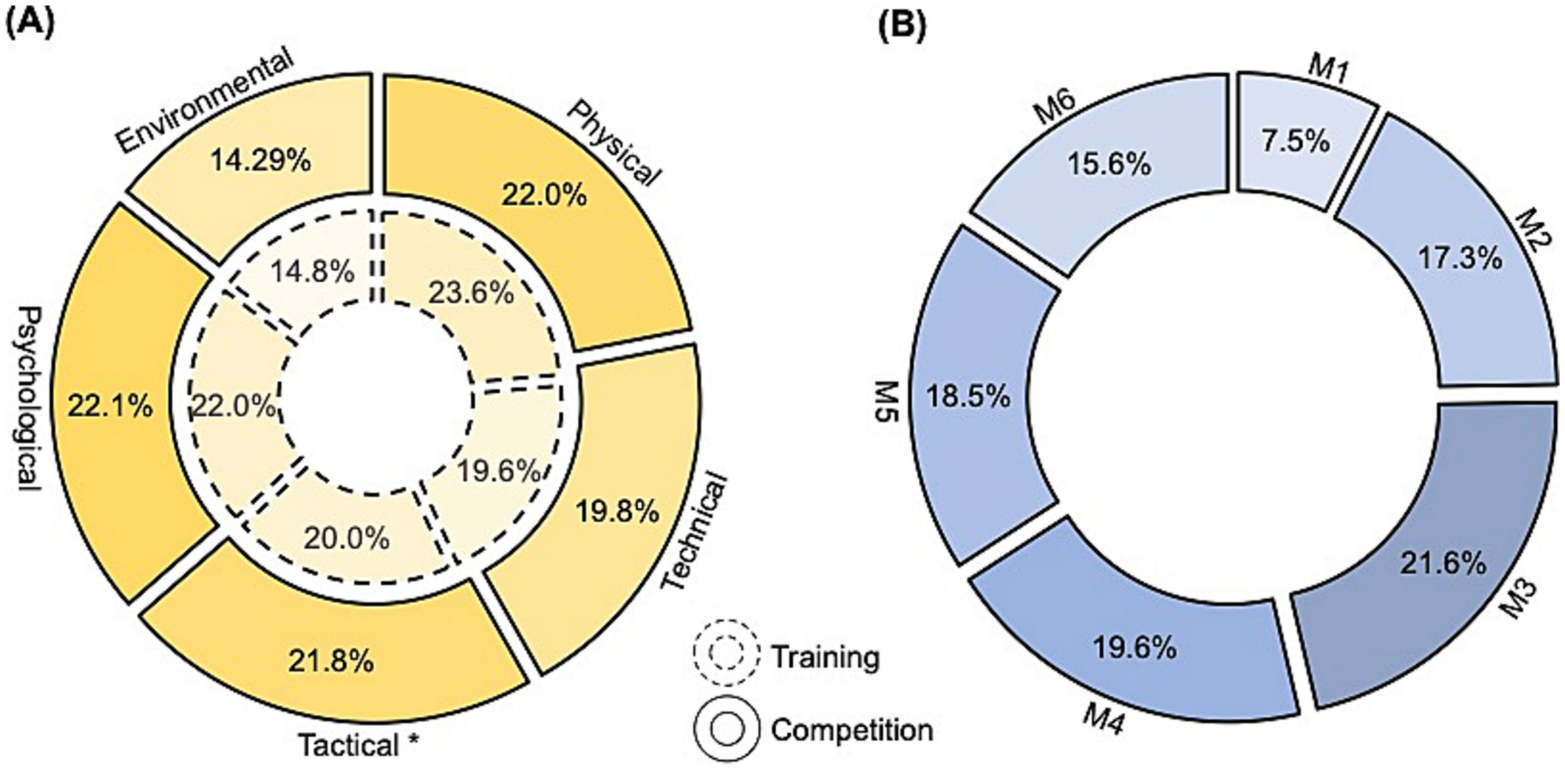
Perceived contributors to the overall mental fatigue inducement process. **(A)** Contributions from five general workloads; the inner circle means in the training context, and the outer circle means in the competition context. **(B)** Contributions from six general fencing combat moments in fencing bouts; M1, posture preparation; M2, step movement; M3, timing decision-making; M4, attack; M5, defense; M6, riposte; *, significant differences of contributions for competition, compared with the contribution in the training context.

From the perspective of different workload factors that might contribute to the overall mental fatigue after fencing activities, significant differences between training and competition contexts were only observed in the tactical factor (see Figure 1A, 20.0% in training vs. 21.8% in competition, *p* = 0.01), while other factors showed no significant changes (physical, *p* = 0.07; technical, *p* = 0.77; psychological, *p* = 0.84; environmental, *p* = 0.54).

From the perspective of the combat execution moments of the fencing bouts in Figure 1B, respondents assessed the contributions of each moment in inducing the overall mental fatigue, with rates indicating that the accumulative moments of timing decision-making and attack execution (21.6% vs. 19.6%, *p* = 0.22) have the greatest contribution.

## 4 Discussion

The results derived from this cross-sectional survey provided insights into the subjective experience of mental fatigue and an understanding of its potential inducement process among (inter)national level fencers. The survey highlighted an elevated mental fatigue perception on match days due to significantly higher tactical load compared to training days. The above-moderate level of MVAS was only retrospectively reported by fencers when the competition context switched from the pool stage to the direct elimination stage. The accumulative timing decision-making process and offensive moments were reported to contribute most to the mental fatigue inducement.

### 4.1 Representativeness of the respondents

Respondents demonstrated high proficiency with national or international experience, representing Tiers 3–5 (McKay et al., 2021) of the sport. The active junior (18–20 years) and senior (> 20 years) fencers aligned with the relatively young age focus in fencing research (Harmer, 2019). The female/male distribution of respondents was consistent with another survey conducted on the British fencing community, with a similar 40% being female (Morris et al., 2022). The relatively balanced distribution enhances the generalization of this survey’s findings. Nevertheless, no evidence of discipline- and gender-specific differences was found by monitoring subjective fatigue and mental effort through simulated combats in elite fencing (Varesco et al., 2023). These findings supported analysis and interpretation of the survey from a unified perspective of the elite fencing population including current and recently retired athletes.

### 4.2 Perception of mental fatigue in fencing

#### 4.2.1 General attitudes to mental fatigue

The findings indicate fencers have a nuanced perception of the concept of mental fatigue. More than half expressed uncertainty in distinguishing mental fatigue from physical fatigue following fencing practice. This aligns with previous research indicating that athletes and staff may find it “hard to decouple” mental and physical fatigue (Russell et al., 2019). This also aligns with the self-evaluated higher contribution of physical workloads to overall mental fatigue in training sessions. As fatigue is multifactorial in nature (Halson, 2014), it manifests as a dynamic event involving subjective, mental, behavioral, neural, and physiological processes that interact over time and across contexts (Tran et al., 2020).

The survey responses indicate that fencers recognized the negative effects of mental fatigue, yet, the community was also aware of the potential benefit of inducing mental fatigue in training. Indeed, it offers increased opportunities for learning, adaptation, management, and the development of tolerance and resilience (Russell et al., 2024). Long-term training adaptation could help more experienced athletes have greater resistance to mental fatigue and the potential negative effects on subsequent performance (Russell et al., 2019). The perception among fencers paves the way for practitioners to incorporate more cognitive training elements into physical practice and the overall training prescription, facilitating the potential application of sport-specific elements of brain endurance training (Roelands and Bogataj, 2024) in fencing sports to benefit the performance level.

#### 4.2.2 Feelings associated with mental fatigue

Fencers expressed above-normal tiredness, negative alterations in emotional states and psychomotor functions when mentally fatigued. Consistent with the laboratory findings, the mental fatigue state has been proven to increase the perceived effort (Pageaux and Lepers, 2016). In certain cases, mental fatigue has been associated with changes in motivational states or negative feelings such as anxiety, frustration, and boredom, with the person experiencing aversion to continue performing tasks (Diaz-Garcia et al., 2021). An effort-reward imbalance may occur (Boksem and Tops, 2008) in mentally fatigued fencers, which stimulates the mental inhibition system to avoid disruption of normal homeostasis (Salihu et al., 2022). This protective mechanism drives thoughts of disengagement, discomfort, and reduces enthusiasm from fencing activities, which is consistent with other elite athlete’s most frequently identified behavioral descriptors in mental fatigue (Russell et al., 2019).

#### 4.2.3 Contextual differences in mental fatigue perception

The retrospectively self-reported MVAS data showed noticeable variations between the training and competitive contexts. In regular training sessions and the initial pool stage of the match day, the MVAS scores were rated lower compared to the context of direct eliminatory stage, where a nearly moderate level increase was observed. The high intermittency of fencing practice, characterized by the short duration of assaults within bouts, and recovery time settings between bouts and games, likely prevented the mental fatigue perception reaching a maximal level (Turner et al., 2017; Varesco et al., 2023). Meanwhile, the fencers’ distinct feelings about mental fatigue in different contexts are supported by longitudinal evidence from other sports, a similar elevation of mental fatigue after successive eliminatory matches has been reported during the professional padel tournament (Diaz-Garcia et al., 2021). The mental fatigue also increased from the regular season to the playoffs in semi-professional soccer (Díaz-García et al., 2023), and during the pre-season training phase when the season approached (Russell et al., 2022b). On match days, where multiple matches or bouts of competition (such as tournament style play) occur, there appears to be an accumulation of mental fatigue (Varesco et al., 2023). This may also be attributed to match difficulties, with more tactical demands and psychological workloads as the match and tournament progress towards the finals (Roi and Bianchedi, 2008; Diaz-Garcia et al., 2021). These fencers recognized the presence of mental fatigue across training and competition, with the perception of mental fatigue tending to be elevated in the later stages of competition. The potential carry-over of mental fatigue from earlier matches and training schedules, needs further investigation during fencing competitions over a longer time frame.

#### 4.2.4 Other factors perceived to relate to mental fatigue

Besides training and competition, other daily cognitive activities and well-being factors were also suggested to play a role in mental fatigue perception, aligning with prior research findings (Russell et al., 2019; Abbott et al., 2020). Fencers recognized that individuals’ health factors (i.e., sleep, diet, and emotion) and competition-oriented elements (i.e., match environment and preparation) synergistically affected mental fatigue. Indeed, sleep has been previously found to have a strong correlation with mental fatigue among professional soccer players during an under-23 Premier League season (Abbott et al., 2020). Inadequate sleep and nutrition, as well as negative emotions, can lead to impairments in specific brain function related to the specific brain regions or networks and aspects of athletic performance, which in turn may negatively affect fencers’ effort and fatigue perception (Mata et al., 2021).

Fencers less strongly perceived their external commitments such as managing relationships, extra work and education, family, transportation, and media to induce mental fatigue. It is contrary to other studies that primarily focused on team based sports (i.e., netball, football), where the athletes and staff recognized such external commitments as a higher order theme related to the presence of mental fatigue (Russell et al., 2019; Thompson et al., 2021). The differences in findings demonstrate that national-level fencing environment may provide unique stimulus. Commonly, elite fencers engage in more personalized drills at a closed-off training base, competition-oriented training and targeted fencing events occupy their entire careers, thus they may be arguably more prepared individually to manage mental fatigue. This structure differs from the majority of professional team sports which have continuous weekly home-away matches in seasons with frequent transportation, high media attention, and more intra-teamwork. Additionally, athletes’ personality types and psychological profiles may differ in one-on-one combat sports. National Foil fencers’ typical traits were profiled as independent and reserved (Roi and Bianchedi, 2008). Plausibly, elite fencers focus on themselves and associate the mental fatigue with a distinct, personal concern related to health and competition. These individual health factors (e.g., sleep, diet, emotional state), their relationships with mental fatigue post-fencing activities, and the interactions with fencer’s daily routines, need to be explored in future studies.

### 4.3 Understanding fencing-specific contributors to mental fatigue inducement

This study introduced novel perspectives on the mental fatigue inducement process, comparing general workload factors and combat moments that fencers rated to contribute to overall perception of mental fatigue. Significantly higher tactical loads accounted for the elevated MVAS post-competition compared to post-training, indicated by retrospective self-reports. The accumulative execution of timing-related decision-making, particularly in attacks, was rated as the most effortful moment that induced mental fatigue during the match.

Fencing is inherently a more offensive activity, in which a single attack can be initiated and completed within a very short time, often requiring immediate and decisive actions (Aquili et al., 2013). Fencers must continuously adapt their tactical strategies, execute precise techniques rapidly, and manage psychological load under competitive pressure (Gutiérrez-Davila et al., 2019; Doron and Martinent, 2021). Although movement speed is important, a key ability is to recognize the best time for starting an attack in response to the opponent’s actions (Roi and Bianchedi, 2008). This may explain the higher contribution of tactical loads to mental fatigue in the one-on-one competitive context. Adjustments to the tactical components of in-season training should be considered, along with the implementation of strategies to manage mental fatigue following intensive tactical sessions in the pre-competition period.

In fencing, cognitive skills such as visual–spatial attention, discrimination, and decision-making are essential to score (Varesco et al., 2024). Fencers perceived that the accumulative moments of timing decision-making contributed most to the mental fatigue inducement. Effective decision-making in fencing involves the allocation of cognitive resources to internal or external stimuli, the rapid analysis of the opponent’s subtle cues from body language (e.g., arm and leg span), previous actions, distance, and sword information (Turner et al., 2017), predicting and selecting appropriate responses, all of which demand substantial mental loads (Varesco et al., 2023). Based on these findings, future studies could design and test representative mental fatigue-inducing tasks and scenario-based simulations for fencing-specific research and training practice with high ecological validity.

### 4.4 Strengths and limitations

The current survey showed practical value for future research and practice by capturing direct perceptions and understanding of the mental fatigue inducement from representative fencing athletes and practitioners. However, the cross-sectional and exploratory approach to the study limits the inferred causality between mental fatigue and fencing performance aspects. The participant group in this survey is representative of current and recently retired Chinese (inter)national-level fencing athletes, accordingly caution should be taken in extending or generalizing findings beyond this population.

### 4.5 Future directions for research and practice

This study forms a strong foundation for research and practice on mental fatigue in fencing sports. Future studies should include a diverse sample of fencers and incorporate long-term multifactor observations to validate and extend the current findings, for example by capturing real-time behavioral and neurophysiological data. Longitudinally tracking over different phases of training and matches could provide further evidence on the reported contextual difference and temporal/accumulative profiles of fencing-specific mental fatigue. Practitioners should place greater awareness on understanding the mental states of fencers, particularly emphasizing the role of individual health factors, pre-competition preparation quality, and the tactical component in training to mitigate mental fatigue and enhance performance. Additionally, to better understand the contributors to mental fatigue, it is important to examine how specific in-game scenarios, such as offensive situations and timing-related decision-making tasks in fencing bouts, impact performance when applied to deliberately induce mental fatigue. This will further support mental fatigue studies with greater ecological validity and will enhance mental fatigue-based training and management in practical settings.

## 5 Conclusion

This cross-sectional survey collected the mental fatigue perceptions and general understanding of the mental fatigue inducement process in fencing from an (inter)national athletes level perspective. It highlighted the contextual differences of mental fatigue (training vs. competition), and the contributions of timing decision-making and offensive moments to the mental fatigue inducement in fencing. Furthermore, mental fatigue was perceived higher when fencing context switched from the tournament pool stage to the direct elimination stage. Individuals’ health conditions and competition-oriented elements were rated as the most significant synergistic factors in the perception of mental fatigue, while the external commitments were rated lower. These findings emphasize the presence of mental fatigue in fencers and encourage researchers and practitioners to effectively identify and deliberately manage mental fatigue.

## Data Availability

The raw data supporting the conclusions of this article will be made available by the authors, without undue reservation.

## Footnotes

^1^ www.sojump.com
