# Supplementary material for: Understanding of mental fatigue in elite fencing sports: perspectives from Chinese national level fencers
Source: Front Psychol. 2025 Apr 14;16:1512326. doi: 10.3389/fpsyg.2025.1512326 (PMC12035770; doi:10.3389/fpsyg.2025.1512326)
Supplement: Supplementary file 1 [file Data_Sheet_1.pdf]

Questionnaire 问卷:

Understanding the Perception, the Generation, and the Contributors of Mental Fatigue in Fencing - Evidence from the Chinese National Fencing Team

中国国家级击剑运动员对于击剑运动中的心理/精神疲劳的综合感知和其形成过程的理解

Front Page.

Informed consent 知情同意书

Page 2.

Background reading 背景材料阅读

Page 3.

Part A. Individual basic information: (Demographic info., fencing identity, fencing level, fencing experience) 基本信息（身份，项目，水平，经验）

1. The current role(s) that fit you? (Multi-select) 您现在的身份是？请选择适用的身份描述（可多选）：

Active fencing athlete 现役运动员
Retired fencing athlete 退役运动员
Fencing coach 教练员
Fencing referee 裁判员
Performance staff 队伍表现人员
Other practitioner with fencing experience 其他有击剑经验的服务人员 please indicate your current role in the team __（展开填写/选择相关的其他角色，比如科研人员/队医/物理治疗师/心理师/数据分析师）

2. What is your age? (years) 您的年龄是？

__

3. What gender do you identify as? 您的性别是？

Man or Male 男
Woman or Female 女
Not-binary 非二元的性别
Prefer not to say 不想告知

4. What specific fencing event are you participating/serving now? (single-select for active athletes, multi-select for others) 您目前从事的击剑具体项目是？（对现役运动员单选，其他可多选）

Female Foil 女子花剑
Male Foil 男子花剑
Female Epee 女子重剑
Male Epee 男子重剑
Female Sabre 女子佩剑
Male Sabre 男子佩剑
Haven't determined 未定项

5. Your total years of experience in fencing sport? 您击剑运动从业经验迄今为止总计多少年了？

—

6. Your total years of national-level team experience? 您在国家队的经验迄今为止总计多少年了？

—

7. Your total years of international-level competition experience? 您的国际比赛经验迄今为止总计多少年了？

—

8. Your highest level of fencing event participation during your career? 您生涯所经历的最高级别击剑比赛是？

Olympic games 奥运会
Other world-level tournaments 其他世界级别
Asian level 亚洲级别
National level 国家级别
Others 其它

Page 4.

Part B. Perception of mental fatigue in fencing：(+ Definition from literature + descriptors from team sports (Russell et al., 2019)) （材料阅读，阅读来自文献的定义+描述词）

1. To what extent do you think you are able to discriminate between physical fatigue and mental fatigue after fencing practice? 您能区分击剑运动后产生的身体层面的疲劳和心理层面的疲劳吗？

Not at all 根本不能
Somewhat 稍微能
Largely 很大程度能

2. Do you believe a training-session-long or match-round-long fencing activity will cause mental fatigue? 您认为击剑运动会引起心理疲劳吗？

Not at all 根本不会
Somewhat 稍微有点
Largely 很大程度会

3. If yes, please retrospectively evaluate: to what extent your mental fatigue is after a typical comprehensive training session? (0-100%, none at all - maximal, Sliding VAS) 如果答案是“会”，请评估一场典型的击剑综合训练课后您所感知到的心理疲劳的程度是：0%为一点也没有，100%为极大 （滑动 VAS 量表）。

—

4. If yes, please retrospectively evaluate: to what extent your mental fatigue is after pool stage competitions on an official match day with equivalent opponents? (0-100%, none at all - maximal, Sliding VAS) 如果答案是“会”，请评估一场官方击剑比赛中面对同水平对手的小组赛后您所感知到的心理疲劳的程度是：0%为一点也没有，100%为极大 （滑动 VAS 量表）。

—

5. If yes, please retrospectively evaluate: to what extent your mental fatigue is after direct elimination competition on an official match day with equivalent opponent? (0-100%, none at all - maximal, Sliding VAS) 如果答案是“会”，请评估一场官方击剑比赛中面对同水平对手的直接淘汰赛后您所感知到的心理疲劳的程度是：0%为一点也没有，100%为极大 （滑动 VAS 量表）。

—

6. How do you define mental fatigue in fencing? (open-ended question, responses can be written/recorded with several keywords as descriptors or sentence about associated feelings) 请您用几个关键词句或相关感受来描述您在击剑运动中产生的心理疲劳感。（开放回答，若干描述的词句，打字或录音回答）

—

7. To what extent do you feel the mental fatigue can affect fencing performance? (-100%-0: negative effect; 0-100 % positive effect; sliding two times VAS) 您觉得心理疲劳会在何种程度上影响击剑运动表现？（-100%到 0 为损害程度，0 到 100%为提升程度，滑动两次 VAS 量表）

Positive effect __

Negative effect __

8. Aside from the fencing training and matches, are there any external factors which you think will interact with your perceptions of mental fatigue? (Multi-select) 除了击剑训练和比赛本身，您觉得最可能影响您击剑比赛后心理疲劳感的外部因素有哪些？（多选，补充填写）

Sleep 睡眠
Daily lifestyle 日常生活方式

Diet 饮食
Emotion 情绪
Interpersonal relationship 人际关系
Match location 比赛地点
Field environment 比赛环境
Preparation duration 备赛期时长
Transportation 交通
Academic requirement 学习要求
Non-competition work arrangement 非比赛工作安排
Family 家庭
Media 媒体
Others (please add) __ 其他（请补充）

Page 5.

Part C. Understanding of mental fatigue inducement process in fencing：

(+ 5 general workload factors in fencing activities: physical, technical, tactical, psychological, and environmental load contributors;
+ 6 criticial combat moments in a fencing bout: posture preparation, step movement, timing decision-making, attack, defense, riposte)

1. If entire workload in fencing activities is categorized into 5 general workload factors: physical, technical, tactical, psychological, and environmental load factors. Please respond below with to what extent you believe each of these factors will contribute to your overall mental fatigue feeling after an official fencing competition with equivalent opponent. Please rate the contribution respectively. (Rating 0-100%, sliding VAS, respectively) 如果将击剑活动中的整体工作负荷按照表现的影响因素分为五类：体能，技术，战术，心理，场外因素；您认为它们对一场势均力敌的官方击剑比赛后所产生的心理疲劳的影响程度分别是多少？请评估其影响的程度（5 次分别评估影响程度 0-100%，滑动 VAS）。

Physical factors 体能因素 __
Technical factors 技术因素 __
Tactical factors 战术因素 __
Psychological factors 心理因素 __
Environmental factors 外部环境因素 __

2. If entire workload in fencing activities is categorized into 5 general workload factors: physical, technical, tactical, psychological, and environmental load factors. Please respond below with to what extent you believe each of these factors will contribute to your overall mental fatigue feeling after a typical comprehensive fencing training session. Please rate the contribution respectively. (Rating 0-100%, sliding VAS, respectively) 如果将击剑活动中的整体工作负荷按照表现的影响因素分为五类：体能，技术，战术，心理，场外因素；您认为它们对典型的击剑综合训练课后所产生的心

理疲劳的影响程度分别是多少？请评估其影响的程度（5 次分别评估影响程度 0-100%，滑动 VAS）。

Physical factors 体能因素 __
Technical factors 技术因素 __
Tactical factors 战术因素 __
Psychological factors 心理因素 __
Environmental factors 外部环境因素 __

3. According to the execution time, if the fencing bout is divided into 6 critical combat moments: posture preparation, step movement, timing decision-making, attack, defense, riposte. Please respond below with to what extent you believe each of these moments will contribute to your overall mental fatigue feeling after an official fencing competition with equivalent opponent. Please rate the contribution respectively. (Rating 0-100%, sliding VAS, respectively) 如果按照不同的执行时间将一次击剑回合分为六个关键时刻：准备姿态，步伐移动，时机决策，进攻执行，防守执行，还击执行。您认为它们各自对势均力敌的官方击剑比赛后所产生的心理疲劳的影响程度分别是多少？请评估其影响的程度（6 次分别评估影响程度 0-100%，滑动 VAS）。

Posture preparation 准备姿态 __
Step movement 步伐移动 __
Timing decision-making 时机决策 __
Attack execution 进攻执行 __
Defense execution 防守执行 __
Riposte execution 还击执行 __

Confirmation and submission 确定全部答题并且提交至问卷星网页终端。
